# Neural Abnormalities Associated with Generalized Anxiety Disorder: A Meta-Analysis of Functional Magnetic Resonance Imaging Activation Studies

**DOI:** 10.1192/j.eurpsy.2023.971

**Published:** 2023-07-19

**Authors:** S. K. Kahlon, Z. Ali, E. Pritchard, S. Saravia, C. Baten, A. M. Klassen, J. H. Shepherd, G. Zamora, J. Jordan, M. Duran, S. L. Santos, D. W. Hedges, J. P. Hamilton, M. D. Sacchet, C. H. Miller

**Affiliations:** 1 Department of Biology, Department of Psychology; 2 Department of Psychology, California State University, Fresno; 3 Department of Psychology, Brigham Young University, Provo, United States; 4 Department of Biomedical and Clinical Sciences, Linköping University, Linköping, Sweden; 5 Meditation Research Program, Department of Psychiatry, Massachusetts General Hospital, Harvard Medical School, Boston, United States

## Abstract

**Introduction:**

Generalized anxiety disorder (GAD) is a highly prevalent mental illness that is associated with clinically significant distress, functional impairment, and poor emotional regulation. Primary functional magnetic resonance imaging (fMRI) studies of GAD report neural abnormalities in comparison to healthy controls. However, many of these findings in the primary literature are inconsistent, and it is unclear whether they are specific to GAD or shared transdiagnostically across related disorders.

**Objectives:**

This meta-analysis seeks to establish the most reliable neural abnormalities observed in individuals with GAD, as reported in the primary fMRI activation literature.

**Methods:**

We conducted an exhaustive literature search in PubMed to identify primary studies that met our pre-specified inclusion criteria and then extracted relevant data from primary, whole-brain fMRI activation studies of GAD that reported coordinates in Talairach or MNI space. We then used multilevel kernel density analysis (MKDA) with ensemble thresholding to examine the differences between adults with GAD and healthy controls in order to identify brain regions that reached statistical significance across primary studies.

**Results:**

Patients with GAD showed statistically significant (α=0.05–0.0001; family-wise-error-rate corrected) neural activation in various regions of the cerebral cortex and basal ganglia across a variety of experimental tasks.

**Conclusions:**

These results inform our understanding of the neural basis of GAD and are interpreted using a frontolimbic model of anxiety as well as specific clinical symptoms of this disorder and its relation to other mood and anxiety disorders. These results also suggest possible novel targets for emerging neurostimulation therapies (e.g., transcranial magnetic stimulation) and may be used to advance our understanding of the effects of current pharmaceutical treatments and ways to improve treatment selection and symptom-targeting for patients diagnosed with GAD.

**Disclosure of Interest:**

None Declared

